# Analysis of independent risk factors for postoperative stress ulcers in high-altitude areas

**DOI:** 10.3389/fmed.2026.1760281

**Published:** 2026-05-04

**Authors:** Dong-Dong Meng, Wen-Jin Xie, Li-Juan Chen, Ling-Fang Zhao, Ya-Dong Li

**Affiliations:** 1 Department of Urology, 940th Hospital of the Joint Service Support Force of the Chinese People's Liberation Army, Lanzhou, China; 2 Breast Surgery Department, 940th Hospital of the Joint Service Support Force of the Chinese People's Liberation Army, Lanzhou, China; 3 The First Outpatient Department of the 940th Hospital of the Joint Logistics Support Force of the Chinese People's Liberation Army, Lanzhou, China; 4 Department of Critical Care Medicine, 940th Hospital of the Joint Support Force of the Chinese People's Liberation Army, Lanzhou, China

**Keywords:** anxiety levels, high altitude, history of digestive ulcer, intraoperative blood loss, risk factors, stress ulcer, surgery

## Abstract

**Background:**

Surgical procedures can increase the incidence of stress ulcers. This study aims to investigate the occurrence of postoperative stress gastric ulcers in a high-altitude environment and to identify independent risk factors through multivariate logistic regression analysis.

**Methods:**

A retrospective analysis was conducted on data from 106 patients who underwent surgical treatment at a medical institution located above 4,000 meters in altitude between January 2023 and December 2024. Collected information included gender, age, alcohol consumption, smoking habits, anxiety levels, depressive status, history of digestive ulcer, type of anesthesia, surgical site, intraoperative blood loss, and duration of surgery.

**Results:**

Among the 106 surgical patients, 33 developed postoperative stress ulcers. Univariate analysis revealed significant differences between the two groups in terms of anxiety levels, history of digestive ulcer, type of anesthesia, surgical site, intraoperative blood loss, and duration of surgery. Multivariate logistic regression analysis indicated that anxiety levels, history of digestive ulcer, and intraoperative blood loss were independent risk factors for postoperative stress ulcers.

**Conclusion:**

In high-altitude areas, the incidence of postoperative stress ulcers significantly increases. The level of anxiety, a history of peptic ulcer disease, and the amount of intraoperative blood loss are important risk factors for postoperative stress ulcers. Preoperative psychological assessment and support should be implemented, surgical trauma should be minimized, and perioperative management should be strengthened to reduce the incidence of postoperative stress ulcers.

## 1 Introduction

Stress ulcers (SU) refer to the acute erosion and ulceration of the gastrointestinal mucosa that occurs in the body under various severe traumas (1), critical illnesses (2), and other stress conditions (3). In extreme cases, SU can lead to complications such as major bleeding (4) or perforation (5). Under fiberoptic gastroscope, SU patients may exhibit gastric mucosal erosion, ulcers, and diffuse bleeding. This type of lesion typically affects only the superficial layers of the digestive tract mucosa and often heals spontaneously within a short period (6).

SU are a common complication following surgical procedures (7), and their primary causes include oxidative stress, excessive gastric acid secretion, *helicobacter pylori* infection, psychological stress and trauma (8). Being at high altitude can reduce visceral blood flow and oxygen levels, leading to hypoxia and oxidative stress, which in turn can damage the gastrointestinal barrier (9). In addition, hypoxia exposure can exacerbate oxidative stress and inflammatory reactions caused by *helicobacter pylori* (Hp) infection, intensify gastric mucosal inflammation and damage, and increase the incidence of SU (10). Multiple studies have shown that intestinal permeability significantly increases in high-altitude environments (11). Concurrently, due to bacterial translocation, the gastrointestinal tract undergoes inflammatory responses at high altitudes, characterized by increased levels of inflammatory markers and indicators of gastrointestinal cell damage (12, 13).

Although previous studies have identified several risk factors associated with SU in high-altitude settings (14), the risk factors for patients undergoing surgery in this environment developing SU have not been fully explored. Therefore, this study aims to analyze data from patients who have undergone surgery in High-Altitude Areas to identify independent risk factors for postoperative SU. By doing so, we hope to improve the quality of postoperative care and enhance patient recovery outcomes in these challenging environments.

## 2 Methods

### 2.1 Participants

This study employed a retrospective cohort design, encompassing adult patients who underwent surgical procedures at the Ritu County People’s Hospital with an altitude of 4,300 meters in Ali Region, Tibet, China, from January 2023 to December 2024. All patients included in the study were aged 18 years or older and were followed up for more than one week post-surgery. Patients with pre-existing active gastric or duodenal ulcers were excluded from the analysis. The final sample size for analysis comprised 106 patients. Informed consent was obtained from all participants prior to their surgical procedures, and the study protocol was approved by the institutional ethics committee, ensuring compliance with ethical standards throughout the research process.

Data were collected through the electronic medical records system and encompassed the following information: gender and age; alcohol consumption and smoking status; anxiety levels assessed using the Generalized Anxiety Disorder 7-item Scale (GAD-7) and depressive symptoms evaluated using the Patient Health Questionnaire 9-item Scale (PHQ-9); presence of a history of digestive ulcers; type of anesthesia, surgical site, intraoperative blood loss, and duration of surgery; and postoperative stress ulcer diagnosis. For standardized assessment and variable classification, the following clear definitions were formulated: (1) GAD-7 and PHQ-9 scales were completed by patients 12–24 h preoperatively under the guidance of trained nurses, under uniform quiet ward conditions to ensure consistency. The GAD-7 and PHQ-9 scales have been validated in high-altitude populations with good reliability; (2) Abdominal surgery is strictly defined as non-gastrointestinal abdominal surgery. To avoid confounding direct mucosal injury, all major gastrointestinal operations (such as gastrectomy and duodenectomy) were completely excluded. Eligible procedures included abdominal wall hernia repair, gynecological surgery, and urological surgery; (3) Alcohol consumption was categorized as a dichotomous variable: regular drinking was defined as consistent alcohol intake (≥3 times per week, with ≥50 mL liquor, ≥300 mL beer, or ≥100 mL wine per occasion) for more than 6 months, while non-drinking referred to no alcohol consumption or occasional intake (<3 times per month). All study data were collected and classified strictly in accordance with the above definitions. Postoperative stress ulcers (SU) are defined as gastric/duodenal mucosal injuries occurring within 7 days after surgery, diagnosed in accordance with the guidelines of the Chinese Journal of Internal Medicine and the American College of Gastroenterology (ACG). Given the objective medical resource limitations at the study site (Ritu County People’s Hospital), professional endoscopic equipment and specialized technical personnel are lacking, precluding gold-standard endoscopic diagnosis. To ensure accuracy and reduce misclassification bias, all SU diagnoses were independently reviewed by two senior physicians, with discrepancies adjudicated by a chief physician. Transient nausea or vomiting within 48 h postoperatively was excluded to avoid false positives. Therefore, the diagnosis of postoperative SU is based on typical clinical manifestations combined with exclusion diagnosis, with the following criteria: (1) Presence of upper gastrointestinal hemorrhage (hematemesis, melena, positive fecal occult blood) or persistent severe epigastric pain; (2) Clear surgical stress as the etiological trigger; (3) Exclusion of other potential etiologies (e.g., acute gastroenteritis) via comprehensive clinical evaluations and routine laboratory tests. Isolated nausea or vomiting, especially transient symptoms occurring within 48 h postoperatively, are excluded from the diagnostic indicators for SU.

### 2.2 Statistical analysis

Statistical analyses were performed using SPSS 26.0 (IBM Corp., Armonk, NY, USA). Univariate analysis was performed to screen for potential risk factors for postoperative stress-induced gastric ulcers, with independent samples *t*-tests and Chi-square tests applied for distinct variable types based on their distribution characteristics. For continuous variables (e.g., age, GAD-7 score, surgical duration), independent samples *t*-tests were used to compare intergroup mean differences between the postoperative SU group and non-SU group. The application of *t*-tests was strictly justified by the fulfillment of two key statistical assumptions: first, the normality of all continuous variables was verified using the Shapiro–Wilk test, with all variables conforming to a normal distribution (all *p* > 0.05); second, the homogeneity of variance for continuous variables was confirmed via Levene’s test (all *p* > 0.05). The Chi-square test was then used for intergroup comparisons of categorical variables (e.g., gender, history of digestive ulcer, type of anesthesia). Variables that were statistically significant in the univariate analysis were included in a multivariable logistic regression model to identify independent risk factors, and adjusted odds ratios (OR) with their 95% confidence intervals (CI) were calculated. To ensure the stability and accuracy of the model, multicollinearity analysis was performed to assess collinearity between variables using the variance inflation factor (VIF), and the goodness-of-fit of the model was evaluated via the Hosmer-Lemeshow test. A *p*-value of less than 0.05 was considered statistically significant.

## 3 Result

### 3.1 Baseline characteristics of participants

A total of 106 patients who underwent surgical procedures were included in this study. Among them, 73 patients (68.87%) did not develop postoperative stress-induced gastric ulcers, while 33 patients (31.13%) did develop such ulcers following surgery. Basic characteristics of the patients, including gender, age, alcohol consumption, and smoking habits, are presented in Table 1.

**Table 1:** Baseline characteristics of participants.

Variables	Postoperative gastric ulcer (%)		Total	X^2^/*t*	*p*
	No (*n* = 73)	Yes (*n* = 33)			
Gender				0.07	0.79
Female	33 (45.21)	14 (42.42)	47 (44.34)		
Male	40 (54.79)	19 (57.58)	59 (55.66)		
Age	29.12 ± 6.31	27.94 ± 5.39	28.75 ± 6.04	0.93	0.35
Drinking				0.87	0.35
No	29 (39.73)	10 (30.30)	39 (36.79)		
Yes	44 (60.27)	23 (69.70)	67 (63.21)		
Smoking				1.10	0.29
No	39 (53.42)	14 (42.42)	53 (50.00)		
Yes	34 (46.58)	19 (57.58)	53 (50.00)		
GAD-7	3.10 ± 4.26	7.70 ± 4.93	4.53 ± 4.94	−4.90	<0.01
PHQ-9	3.93 ± 4.42	4.82 ± 5.19	4.21 ± 4.66	−0.91	0.37
History of gastric ulcer				14.30	<0.01
No	58 (79.45)	14 (42.42)	72 (67.92)		
Yes	15 (20.55)	19 (57.58)	34 (32.08)		
Type of anesthesia				23.83	<0.01
Local anesthesia	20 (27.40)	0 (0.00)	20 (18.87)		
Waist numbness	44 (60.27)	16 (48.48)	60 (56.60)		
General anesthesia	9 (12.33)	17 (51.52)	26 (24.53)		
Surgical site				7.27	0.03
Oral cavity	18 (24.66)	2 (6.06)	20 (18.87)		
Perineum	30 (41.10)	12 (36.36)	42 (39.62)		
Abdomen	25 (34.25)	19 (57.58)	44 (41.51)		
Surgical bleeding volume				36.45	<0.01
Less than 50 mL	54 (73.97)	6 (18.18)	60 (56.60)		
50–100 mL	13 (17.81)	8 (24.24)	21 (19.81)		
Over 100 mL	6 (8.22)	19 (57.58)	25 (23.58)		
Surgical duration (min)	53.99 ± 22.53	70.06 ± 24.40	58.99 ± 24.20	−3.31	<0.01

Univariate analysis revealed significant differences between the postoperative stress-induced gastric ulcer group and the no-stress ulcer group in terms of anxiety levels, history of peptic ulcer disease, type of anesthesia, surgical site, intraoperative blood loss, and duration of surgery. Specifically, patients with a history of peptic ulcers and those who received general anesthesia were more prone to developing stress-induced gastric ulcers. Among patients who underwent abdominal surgery, a higher proportion developed postoperative stress-induced gastric ulcers. Similarly, a greater proportion of patients with higher intraoperative blood loss experienced such ulcers. Moreover, the mean duration of surgery was significantly longer in the postoperative stress-induced gastric ulcer group compared to the no-stress ulcer group. These findings suggest that anxiety levels, history of peptic ulcer disease, type of anesthesia, surgical site, intraoperative blood loss, and duration of surgery are important factors associated with the development of postoperative stress-induced gastric ulcers.

### 3.2 Multivariable logistic analysis

To further identify independent risk factors, we included the variables that were statistically significant in the univariate analysis into a multivariable logistic regression model. The results showed that anxiety levels (as measured by GAD-7 scores), history of peptic ulcer disease, and intraoperative blood loss were independent risk factors for the development of postoperative stress-induced gastric ulcers (Table 2).

**Table 2:** Multivariable logistic analysis.

Variables	b	SE	*z*	Wald χ2	*p*	OR	OR (95% CI)
GAD-7	0.14	0.06	2.26	5.1	0.02	1.15	1.02 ~ 1.29
History of gastric ulcer	2.26	0.73	3.08	9.51	<0.01	9.55	2.28 ~ 40.04
Type of anesthesia	0.77	0.54	1.43	2.05	0.15	2.15	0.75 ~ 6.16
Surgical site	0.46	0.58	0.8	0.63	0.43	1.58	0.51 ~ 4.90
Surgical bleeding volume	0.97	0.47	2.08	4.32	0.04	2.64	1.06 ~ 6.57
Surgical duration (min)	0.01	0.01	0.77	0.59	0.44	1.01	0.98 ~ 1.04

### 3.3 Model collinearity diagnostics and goodness-of-fit assessment

As shown in the Table 3, all variables included in the multivariable analysis had Variance Inflation Factor (VIF) values less than 5, and tolerance levels greater than 0.1, thereby ruling out the issue of multicollinearity among the independent variables.

**Table 3:** Model collinearity diagnostics.

Variables	VIF	Tolerance
PHQ-9	1.24	0.81
History of gastric ulcer	1.22	0.82
Type of anesthesia	1.63	0.62
Surgical site	1.83	0.55
Surgical bleeding volume	2.19	0.46
Surgical duration (min)	1.49	0.67

The Hosmer-Lemeshow goodness-of-fit test was used to evaluate the model’s fit. As indicated in the Table 4, the null hypothesis for this test posits that there is no significant difference between the observed and the fitted values, implying that the model adequately fits the data. The *p*-value obtained from the test is greater than 0.05 (Chi-square = 9.521, *p* = 0.300), which means we do not have sufficient evidence to reject the null hypothesis. Consequently, we can conclude that the model satisfies the Hosmer-Lemeshow test, indicating an acceptable goodness-of-fit.

**Table 4:** Hosmer-Lemeshow test.

χ^2^	*df*	*p*
9.52	8	0.30

## 4 Discussion

The study identified that 33 out of 106 surgical patients developed stress ulcers, representing an incidence rate of 31.1%. To investigate the risk factors for postoperative SU in high-altitude settings, univariate and multivariate logistic regression analyses were performed. The findings revealed significant differences between patients who developed postoperative stress ulcers and those who did not, particularly in terms of anxiety levels, history of peptic ulcer disease, type of anesthesia, surgical site, intraoperative blood loss, and duration of surgery. Further multivariate logistic regression analysis confirmed that elevated anxiety levels, a history of peptic ulcer disease, and greater intraoperative blood loss are independent risk factors for the development of postoperative stress ulcers.

People with anxiety often have higher mental stress. Exposure to stress can lead to changes in the brain-gut interaction, often referred to as the “brain-gut axis,” ultimately contributing to the development of a wide range of gastrointestinal disorders. The primary effects of stress on gastrointestinal physiology include alterations in gastrointestinal motility (15), heightened visceral sensitivity (16), negative impacts on mucosal regeneration and blood flow (17, 18), impairing the gut lining’s ability to heal and maintain its integrity and adverse effects on the gut microbiota, disrupting the balance of the microbial community and contributing to dysbiosis, which may exacerbate gastrointestinal disorders and affect overall health (19). Therefore, it is particularly important to conduct psychological assessments and provide support to patients before surgery in high-altitude areas to reduce anxiety levels and prevent the occurrence of postoperative stress-induced gastric ulcers.

A history of peptic ulcer disease emerged as another independent risk factor for postoperative stress-induced gastric ulcers. Peptic ulcer disease is a chronic disease with a cycle of repeated healing/remission and recurrence (20). Within the healed ulcer scar, the quality of ulcer healing is poor due to immature regenerated tissue and distorted structure. Patients with a history of peptic ulcers have impaired gastric mucosal barrier function and may have poorer healing quality, making them more susceptible to surgical pressure (21). The hypoxic environment in high-altitude areas may exacerbate this damage, weakening the protective mechanisms of the gastric mucosa (9, 14). For these patients, preoperative measures to protect the gastric mucosa should be strengthened, such as the use of proton pump inhibitors (PPIs) or other autoprotective agents, to minimize postoperative complications.

Intraoperative bleeding is the third identified independent risk factor. Massive bleeding means greater surgical trauma and longer surgical time, which not only increases the complexity and risk of surgery, but also increases the risk of stress ulcers (22). At the same time, a decrease in blood volume can affect the blood supply to the gastrointestinal tract, causing local ischemia and affecting the barrier of the gastrointestinal mucosa (23). Surgical bleeding in high-altitude areas can lead to gastrointestinal hypoxia and ischemia, and tissues produce large amounts of reactive oxygen species (ROS), which may cause epithelial cell necrosis and the development and deterioration of mucosal ulcers (24). Therefore, minimizing bleeding during surgery is crucial, and effective hemostatic measures should be taken when necessary. After surgery, closely monitoring the patient’s gastrointestinal status is crucial for timely resolution of any potential complications.

While the type of anesthesia, surgical site, and duration of surgery showed significant differences in the univariate analysis, they were not confirmed as independent risk factors in the multivariable logistic regression analysis. This suggests that these factors may interact with other variables or have relatively minor influences. Nevertheless, selecting appropriate anesthesia and optimizing surgical procedures remain important strategies to reduce the incidence of postoperative stress-induced gastric ulcers (25, 26). For example, regional anesthesia (27) compared to general anesthesia may be more favorable for gastrointestinal recovery, and abdominal surgeries, due to their extensive trauma and longer operation times, require special attention to gastric mucosal protection.

This study adopted a retrospective cohort design based on medical records, which may give rise to information bias due to potential documentation omissions or inaccuracies. Additionally, residual confounding factors may still exist despite multivariable adjustment. Notably, the sample size is limited, which is attributable to the unique conditions of the study site—the Ritu County People’s Hospital, located at an altitude of 4,300 meters in a remote high-altitude pastoral area with a very small resident population, resulting in low surgical service demand. This small sample size compromises statistical power, making it hard to detect low-effect risk factors or complex variable interactions; results in wide 95% CIs for some indicators (e.g., history of peptic ulcer: OR = 9.55, 95% CI = 2.28 ~ 40.04) with reduced estimation precision; and precludes subgroup analyses (e.g., by surgical duration/anesthesia type), potentially masking heterogeneous risk factor characteristics across subgroups.

Additionally, the present study failed to fully incorporate high-altitude region-specific factors and several critical risk factors for SU, and this deficiency is directly attributable to the severe objective medical resource constraints at the study site and the inherent functional positioning of primary county hospitals in China’s border areas. First, regarding the insufficiency in including high-altitude specific factors, Ritu County People’s Hospital, as a primary medical institution in an extremely remote high-altitude border area, is characterized by scarce medical testing equipment and limited clinical detection capacity, which makes it impossible to complete the continuous dynamic monitoring and accurate quantitative detection of multiple high-altitude specific physiological indicators (e.g., hypoxic tolerance, oxidative stress index) and related biochemical markers associated with intestinal barrier function (28). This insurmountable objective medical resource limitation directly leads to the inability to include these high-altitude specific factors in the study for further statistical analysis. Second, for the absence of patients with severe acute traumatic injuries in the study cohort, it should be clarified that as a county-level hospital in China’s border high-altitude area, our medical institution is primarily responsible for the surgical management of minor traumatic injuries for local residents. For patients with severe acute traumatic injuries requiring surgical intervention, such as craniocerebral trauma, abdominal impact injury and fractures, our hospital only conducts emergency triage and referral to higher-level tertiary hospitals and does not perform relevant surgical treatments locally, thus this important subgroup is inevitably excluded from the study. Third, for the long-term use of non-steroidal anti-inflammatory drugs (NSAIDs)—a well-recognized critical risk factor for SU that was not included in the current research indicators—we have formulated a clear research plan to formally incorporate this factor into the standardized research indicator system for systematic investigation and rigorous statistical analysis in our subsequent prospective studies on postoperative SU in high-altitude areas, to make up for this research deficiency (29).

The findings of this study provide important reference points for the prevention and management of postoperative stress-induced gastric ulcers in high-altitude areas. Personalized preventive strategies can be developed based on independent risk factors such as anxiety levels, history of peptic ulcer disease, and intraoperative blood loss, including preoperative psychological interventions, application of gastric mucosal protective drugs, and strict control of intraoperative bleeding. Future research could explore additional potential risk factors, such as nutritional status and genetic background, and verify our conclusions through prospective cohort studies. Moreover, developing a predictive model for postoperative stress-induced gastric ulcers suitable for high-altitude areas would aid in enhancing the scientific accuracy of clinical decision-making.

## 5 Conclusion

In summary, this study demonstrates that in high-altitude areas, the levels of anxiety, a history of gastric ulcer disease, and the amount of surgical bleeding are significant independent risk factors for postoperative stress-induced gastric ulcers. By implementing targeted preventive measures and optimizing perioperative management, the incidence of postoperative stress-induced gastric ulcers can be effectively reduced, thereby improving patient outcomes and quality of life.

## Data Availability

The original contributions presented in the study are included in the article/supplementary material, further inquiries can be directed to the corresponding authors.

## Appendix

### References for the questionnaires

The questionnaires used in this study, the Generalized Anxiety Disorder 7-item Scale (GAD-7) and the Patient Health Questionnaire 9-item Scale (PHQ-9), are well-established, validated instruments that have been published in prior research. Since these are pre-published tools, no supplementary files are required for their inclusion. The references for GAD-7: Spitzer, R. L., Kroenke, K., Williams, J. B., & Löwe, B. (2006). A brief measure for assessing generalized anxiety disorder: The GAD-7. Archives of Internal Medicine, 166(10), 1,092-1097.doi:10.1001/archinte.166.10.1092. The references for PHQ-9: Kroenke, K., Spitzer, R. L., & Williams, J. B. (2001). The PHQ-9: Validity of a brief depression severity measure. Journal of General Internal Medicine, 16(9), 606-613.doi:10.1046/j.1525-1497.200 1.016009606.x.
